# Dietary supplement use among lactating mothers following different dietary patterns – an online survey

**DOI:** 10.1186/s40748-023-00171-3

**Published:** 2024-02-01

**Authors:** Franziska Delgas, Lisa Bitsch, Laura Maria König, Damaris Elisabeth Beitze, Veronika Scherbaum, Maren C. Podszun

**Affiliations:** 1 Institute of Nutritional Medicine, University of Hohenheim, Fruwirthstr.12, 70599 Stuttgart, Germany; 2 Faculty of Life Sciences: Food, Nutrition and Health, University of Bayreuth, Bayreuth, Germany; 3 Department of Clinical and Health Psychology, Faculty of Psychology, University of Vienna, Vienna, Austria; 4 Institute of Nutritional Sciences, University of Hohenheim, Stuttgart, Germany

**Keywords:** Nutritional supplements, Lactation, Breastfeeding, Plant-based diets, Vegan, Vegetarian, Micronutrients

## Abstract

**Background:**

Breastfeeding is important for the healthy growth and development of newborns, and the nutrient composition of human milk can be affected by maternal nutrition and supplementation. In Germany, iodine supplementation is recommended for all lactating mothers, and docosahexaenoic acid (DHA) supplementation is recommended for mothers with inadequate or no fish intake. Vitamin B12 supplementation is required for strict vegans during lactation, and other nutrient supplementation may be necessary depending on the individual's nutritional status. To address the lack of data on dietary supplements used by lactating mothers following a vegetarian or vegan diet, an online survey was conducted in Germany, with a focus on iodine, DHA, and vitamin B12.

**Methods:**

Study participants were asked to report whether they followed specific dietary patterns (omnivorous [OM], vegetarian [VT], vegan [VN]) as well as their use of dietary supplements. Relationships between diets and supplement use were analyzed using chi-square tests.

**Results:**

2054 lactating women were included (1240 OM, 410 VT, and 404 VN) in this analysis. Within OM, VT and VN, at least one dietary supplement was taken by 67.3%, 84.9% and 98.0% respectively (*p* < 0.001). Overall, 53.2% OM, 66.8% VT, 88.4% VN reported taking at least one supplement containing iodine (*p* < 0.001). 54.6% OM, 61.7% VT and 58.2% VN reported supplements containing vitamin B12, while 34.1% OM, 40.2% VT and 38.6% VN mentioned supplements containing DHA (*p* < 0.05).

**Conclusion:**

More than half of the participants reported the use of supplements during lactation with the highest proportion in vegans. However, over one third of the mothers did not report supplementing with iodine, regardless of their dietary pattern and most participants also did not report DHA supplements. It is worrisome that a high number of vegans did not report vitamin B12 supplementation, but this could be partly due to issues with reporting. It is crucial to provide further education to breastfeeding mothers about the importance of taking micronutrient supplements, especially for those following a vegetarian or vegan diet. This will help ensure that mothers and their breastfed infants receive optimal nutrition for a healthy development.

**Supplementary Information:**

The online version contains supplementary material available at 10.1186/s40748-023-00171-3.

## Introduction

The lactation period is a vulnerable time for mother and child and the foundation for healthy development [1]. The nutritional status of a lactating mother partly influences the macro- and micronutrient composition of human milk although details of this complicated process are just being unraveled [2, 3]. During breastfeeding, in most cases the infant's energy and nutrient needs are adequately met by the individually adapted human milk [4–6]. However, an inadequate nutrient status of the mother can lead to developmental and growth deficits in fully breastfed infants [7, 8]. There is an increased need for certain nutrients during lactation, which is why these are defined as critical nutrients [9]. With few exceptions, this additional requirement for nutrients, vitamins and minerals can be covered by a balanced diet with an appropriate choice of foods [7].

There are, however, nutrients for which supplementation either for all lactating women or for women following specific dietary patterns are officially recommended in Germany. One such nutrient is iodine, where reported intake levels in Germany are insufficient [10] and a deficiency can occur even with optimal food choices [11–13]. [10] A good supply of iodine of the mother is important as it directly affects the iodine concentration of breast milk [13, 14]. Iodine is essential for the development of the brain, nervous system and mental abilities [10, 13–15] and iodine deficiency can lead to irreversible developmental disorders [16]. The “Healthy Start – Young Family Network”, a joint initiative by the German Federal Ministry of Nutrition and Agriculture and the German Federal Ministry of Health, therefore recommends a daily supplementation of 100 µg iodine to women without thyroid conditions during the lactation period, in addition to the use of iodized table salt [7].

The network further recommends that breastfeeding women who do not consume fish twice a week should supplement 200 mg docosahexaenoic acid (DHA) per day [7, 17]. Docosahexaenoic acid is one of the omega-3 fatty acids that can only be formed to a small extent by the body and must therefore be supplied via the diet (or dietary supplements) [18, 19]. DHA and EPA are vital for childhood brain and eyesight development. Studies also suggest lower allergy rates in children when mothers had sufficient EPA and DHA during pregnancy and lactation [20].

A vegan diet during pregnancy and lactation is not recommended by the German [7, 21], Swiss [22] and Austrian Dietary Associations [23]. Other societies such as the American Academy of Nutrition and Dietetics state that a vegan diet, given supplementation for B12 is ensured, can sustain healthy growth in infants [24]. Vegan diets during lactation can raise the risk of nutrient deficiency, with vitamin B12 being a key concern as it can only be reliably obtained from animal products and must be supplemented [25–28].

The consequences of an inadequate vitamin B12 supply during lactation, especially for the infant, are demonstrated by several case studies, where serious neurological symptoms occurred [29–32]. Deficiency symptoms usually appear between 4–6 months of age, although irreversible damage can often be avoided with an early diagnosis and immediate treatment with vitamin B12 supplements [30–36]. There are also risks for the mother, including hematological and neurological effects, ranging from fatigue and peripheral neuropathy to sever consequences such as the degeneration of the spinal cord [37]. To ensure the well-being of both the mother and infant, a strictly vegan lactating mother should take a vitamin B12 supplement. Recent data also indicates that mothers on a vegetarian diet may face an increased risk of B12 deficiency without supplements [38]. Besides B12, other critical nutrients in lactating mothers following a strict vegetarian or vegan diet may be protein, riboflavin, vitamin D, calcium, iron, zinc, selenium, DHA/EPA and iodine. In this study we focused on B12, as this needs to be supplemented by every vegan due to the unreliable presence of bioavailable vitamin B12 in plant-based food [39].

A recent, representative cohort study in Germany shows that 94.6% of women in Germany are taking dietary supplements during pregnancy [40]. However, there is currently no data for breastfeeding women in Germany especially stratified by dietary pattern. Therefore, the aim of this study was to investigate the supplementation behavior of breastfeeding women in Germany following different dietary patterns. We focused on the recommended nutrients iodine, which is critical for all lactating women in Germany, DHA, which is only critical with low fish consumption as well as vitamin B12 which needs to be supplemented in a vegan diet.

## Methods

### Study design

This online survey was conducted at the Institute of Nutritional Medicine at the University of Hohenheim. The study collected data on the nutritional and supplementation behavior of lactating women in Germany. Data collection took place in the period from June 01, 2022 to July 16, 2022 (6 weeks). The study protocol was approved by the ethics committee of the University of Hohenheim, Germany. Inclusion criteria were current lactation or lactation within the last six months. Further requirements for participation in the study were a minimum age of 18 years and a permanent residence in Germany. Women with all diets (omnivorous, vegetarian, vegan) were included in the survey. Exclusion criteria were women without a German residence, participants that did not finish the survey, participants younger than 18 and older than 100 years as well as other plausibility checks such as entries of weight below 30 kg and height above 200 cm.

### Procedure

The online survey was conducted with "Unipark" (Tivian). The survey was designed to take about 10–15 min. The questions were arranged in such a way that the answers of the participants could not be influenced by preceding questions to avoid so-called radiation effects (halo effects) and the resulting systematic biases in the responses [41]. It was stated that all questions of the entire survey referred to the youngest child of the test subjects. All participants actively consented to study participation and the use of anonymized data, which was requested by placing a check mark on the start page of the survey. An additional privacy statement was not included, as the survey only collected anonymized data. The participants completed the online-supported questionnaire without the presence of supervisors. They gained access either by scanning the QR code generated by Unipark using a smartphone or tablet, or via the study link also generated in Unipark. The QR code was located on all print media. The study link was used for sharing on social networks or other digital media. Participation in the survey without a computer, tablet or smartphone was not possible. Within the survey, participants answered questions related to the topics of pregnancy, lactation, introduction of solid foods, food consumption (for the used mini food frequency questionnaire see Supplemental methods Table 1), supplementation behavior, anthropometry, and socioeconomic background. As compensation for their participation, all participants received a brochure on healthy nutrition during lactation.

**Table 1:** Criteria for external definition of dietary pattern

Dietary pattern	External definition
Omnivorous	Inclusion of animal products and consumption of meat
Vegetarian	Inclusion of animal products, no consumption of meat
Vegan	No consumption of animal products

### Calculation of sample size

In 2020, a total of 773,144 births were recorded in Germany [42]. Of these, approximately 68% women (*n* = 525,738) fully breastfed their infant for the first four months of life [43]. The nationwide SUSE II survey showed a distribution of 4.5% vegetarian and 0.3% vegan of pregnant and lactating women [44]. Extrapolating from this data, this results in approximately 23,658 vegetarian and 1,577 vegan breastfeeding women per year. Under the expectation of a realistic maximum inclusion rate of a total of 1% of these women in a survey, this would correspond to 237 vegetarian and 16 vegan women. As data on vegan dietary pattern is especially scare we choose to oversample this group aiming at an even distribution of at least 200 participants per dietary pattern (omnivorous, vegetarian, vegan). Therefore, our data is not representative of the distribution of the dietary patterns in the entire society but rather aims to compare the use of dietary supplements by lactating women following different dietary patterns.

### Recruiting strategy

Recruitment was carried out digitally and via the distribution of print media. Instagram was used as a social medium to systematically recruit a specific target group: intentional oversampling lactating mothers on a vegan diet. A systematic hashtag search was used to identify relevant accounts on instagram with a focus on vegan nutrition. Matching accounts were asked to share the call for participation with their followers.

In addition, on-site recruitment took place using 2500 flyers and 50 posters in gynecological and pediatrician practices, via midwives and lactation consultants, family centers, children's stores, bulletin boards in supermarkets in the Stuttgart, Karlsruhe and Tübingen area.

### Measures

#### Demographic information about mother and children

Participants were asked to indicate the number of children they had born (1/ 2/ 3/ 4 or more). They were also asked to indicate the age of the child they were currently breastfeeding (on a scale of 1 to 12 months or older), with the option to indicate that they were currently breastfeeding more than one child, for which they were asked to indicate the ages of the children in months. In addition, demographic information about the mother (age, highest educational attainment, net monthly household income) and anthropometric information (height and current weight) were assessed.

#### Dietary patterns

Dietary patterns were assessed subjectively by asking participants to self-identify as (1) omnivore, (2) ovo-lacto vegetarian (i.e., not consuming meat and fish), (3) lacto-vegetarian (i.e. not consuming meat, fish and egg), (4) ovo-vegetarian (i.e., not consuming meat, fish and milk), (5) pescetarian (i.e. not consuming fish), or (6) vegan (i.e. not consuming animal products). In addition, dietary patterns were objectively defined based on the evaluation of the reported food consumption in the mini FFQ (Supplemental methods table A1) with criteria defined internally by the study group (see Table 1). This was referred to as the so-called external definition and contrasts with the self-reporting of the dietary pattern of the participants at the end of the questionnaire.

#### Information about lactation

Participants indicated whether they were breastfeeding in the past 6 months (yes/no). Participants who indicated to not have breastfed in the past 6 months were excluded from the study. Participants who indicated that they breastfed in the past six months were asked to indicate how they fed their child in the first 12 months of their life, providing a response for each month separately. Response categorizes were as following: (1) fully breastfed, (2) breast milk and breast milk substitute, (3) breast milk substitute, (4) breast milk and complementary food, (5) breast milk substitute and complementary food, (6) breast milk, breast milk substitute and complementary food, (7) complementary food. Participants were informed that full breastfeeding included the use of pumped breast milk. Furthermore, participants were asked whether or not they had already completed breastfeeding and if so at which age on a scale of 1 to 12 months or older. If the option older was chosen a free text field for reporting of age appeared.

#### Intake of supplements

First, participants were asked to indicate whether they took any dietary supplements while breastfeeding (yes/no). If so, they were asked to select products from a list (Supplemental table A2) or provide a free text answer into the text field. Answers were categorized by the research team into intake of a supplement containing iodine, B12 and DHA (each yes/no) for analysis. Free text answers were further analyzed for additional reported micronutrients.

#### Intake of fish

Fish intake was assessed in the mini FFQ (Supplemental table A1) with the following choices of frequency: (1) never, (2) less than once a week, (3) 1–2 times a week, (4) 3–4 times a week, (5) 5–6 times a week, (6) once a day, (7) multiple times a day.

#### Awareness of supplement recommendation

Participants were asked whether they were familiar with the recommendations for taking supplements during lactation by the German Nutrition Society (yes/no).

#### Other information

Participants also provided information on diagnosed allergies and intolerances, changes in dietary intake during pregnancy and lactation, preterm birth, start and mode of complementary feeding, avoidance of food in complimentary feeding and use of supplements for the infants that are not reported in the present analysis.

### Statistical analysis

Statistical analysis was performed using the statistical software "IBM SPSS Statistics Version 27" and "Microsoft Excel, Microsoft Office Professional Plus 2019". Groups were compared regarding demographic characteristics (SES), dietary patterns, lactation patterns and supplement intake using pearson Chi-square; further comparisons between groups regarding demographic characteristics (age, body weight, BMI) were conducted using one-way Analyses of Variance (ANOVA) with Bonferroni-corrected post hoc tests. In addition, multiple logistic regression was conducted to test relationships between dietary patterns, demographic characteristics (age, education, income, being a first-time mother), awareness of supplement recommendation, and supplement intake. For this purpose, education and income were dichotomized. Education was recoded as university degree yes/no; income was split by Germany’s median net household income [45]. The significance level was set at α = 0.05.

## Results

**Fig. 1:**
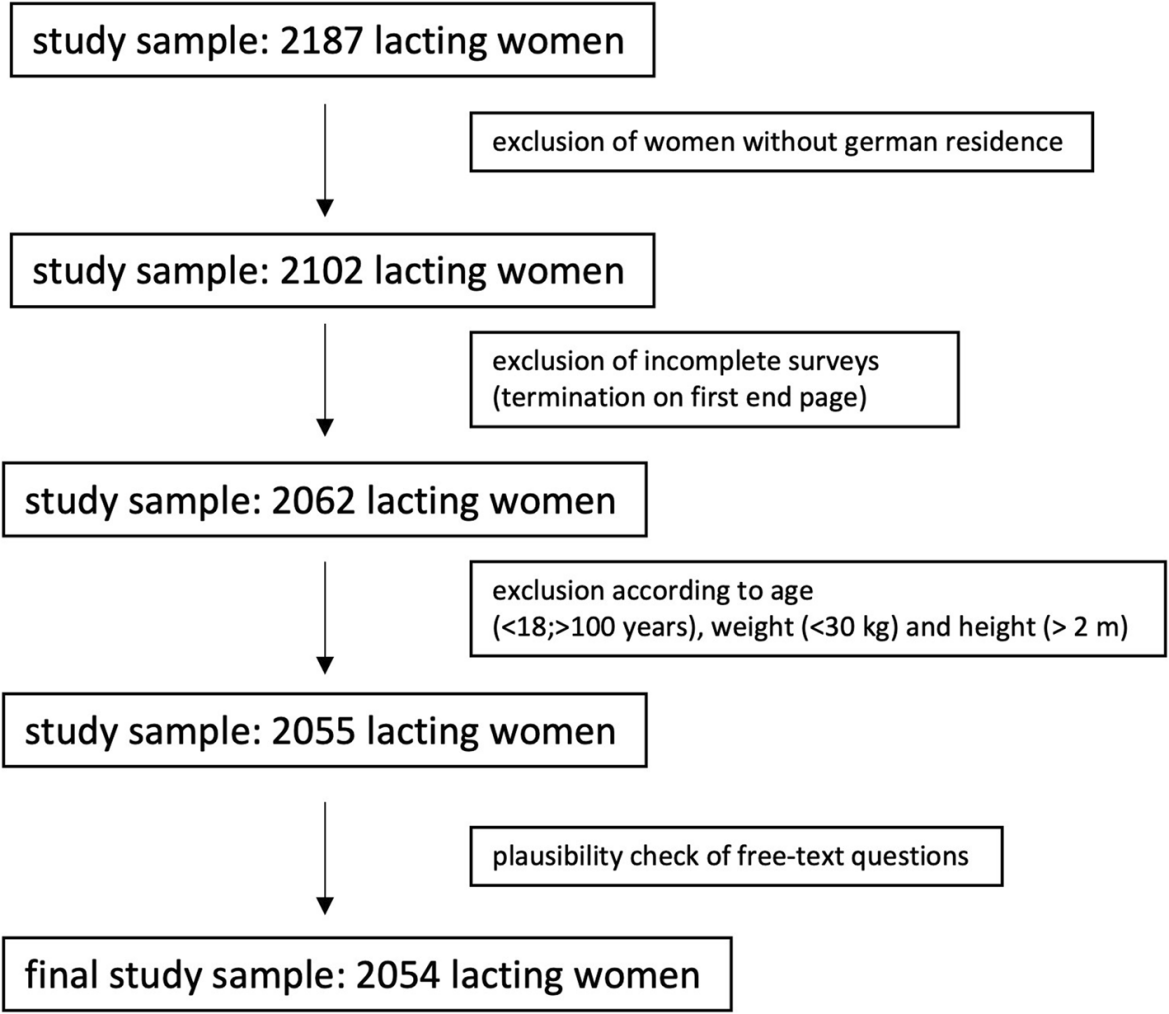
Flow chart of participant exclusion

A total of 2187 participants completed the study. After exclusion of 133 participants, the final study sample included 2054 participants (Fig. 1). Overall, 84.5% of the participants were recruited via social media, 11.0% via personal recommendation, 0.6% via flyers and 3.3% elsewhere. The mean time to complete the survey was 10.5 ± 3.4 min.

### Sample characteristics

2054 women aged 32.2 ± 4.1 years with a BMI of 24.4 ± 4.7 kg/m^2^ were included in this study. The detailed sample characteristics separated by dietary type is shown in Table 2. Body weight, BMI and age were significantly different between the diet groups (*p* < 0.001). Body weight and BMI of the omnivorous participants were both higher than those of the vegan and vegetarian participants. Vegan breastfeeding women were on average one year younger than omnivorous breastfeeding women. In the study population, the mean number of children born was two (2.4 ± 0.7). 92.61% of infants that had already reached 4 months (*n* = 1800) were exclusively breastfeed at this milestone and this decreased to 49.87% at 6 months (*n* = 1642). Full breastfeeding differed between dietary groups significantly only at 6 months, with vegans reporting the highest rates (95.79% and 60.24%). 322 infants were already weaned at the time of the survey and the age of weaning ranged between 1–99 months of age. The average age of weaning was 17.8 months with no significant differences between dietary groups. Approximately one third of the sample (33.6%) reported not to have obtained a university degree, while 66.4% of participants held a university degree. Regarding the monthly net household income, 38.5% reported an income of 2,600 € to < 3,600 € or less; the remaining 61.5% of the sample reported a higher income.

**Table 2:** Characteristics of study population divided by dietary pattern; *n* = 2054

***n* =**	Omnivore	Vegetarian	Vegan	*p*-value (test statistic, df)
	1240	410	404	
Age (years)	32.57 ± 4.00	32.10 ± 4.10	31.51 ± 4.43	< 0.001 (8.44, 2)
Weight (kg)	70.76 ± 14.73	66.04 ± 12.40	66.78 ± 11.84	< 0.001 (24.86, 2)
BMI (kg/m^2^)	25.05 ± 4.99	23.43 ± 4.10	23.31 ± 3.81	< 0.001 (31.32,2)
Number of children (n)	2.46 ± 0.68	2.39 ± 0.68	2.41 ± 0.68	0.17 (1.75, 2)
**Breastfeeding (BF)**				
Full BF 4 months (n, %)^*^n1	987 (91.30)	339 (93.39)	341 (95.79)	0.06, (5.50, 2)
Full BF 6 months (n, %)^*^n2	449 (45.96)	170 (51.05)	200 (60.24)	< 0.001 (22.77, 2)
BF terminated (n)	218	55	49	
Average age of child at weaning (months)^*^n3	16.45 ± 12.22	19.45 ± 10.50	17.89 ± 14.81	0.25 (1.38,2)
**Education and household income**				
Tertiary education (n, %)*	813 (65.6)	285 (69.5)	265 (65.6)	0.32 (2.28, 2)
Income above median (n, %)	808 (65.2)	238 (58)	217 (53.7)	< .001 (19.43, 2)

*n1 = 1800 had reached this milestone (OM 1081, VT 363, VN 356), *n2 = 1642 had reached this milestone (OM 977, VT 333, VN 332), *n3 = 322. *tertiary education refers to a university degree. Values are means ± SDs for metric and n (%) for categorial data; significance was calculated with one way analysis of variance (ANOVA) (age, weight, BMI, number of children, age at weaning) as well as with pearson’s chi-squared test (BF, SES)

### Awareness of supplement recommendations

In this study, a total of 43.82% of the participants reported knowing the supplementation recommendations during breastfeeding by the German Nutrition Society. Omnivorous participants were the least informed (34.76%), followed by vegetarian (45.37%) and vegan (70.05%) participants (*p* < 0.001).

### Dietary patterns

To ensure classification of dietary patterns is accurate we asked participants to self-report their dietary pattern (omnivore, vegetarian, vegan) as well as asked them to fill out a short food frequency questionnaire including food groups with a specific focus on animal products. More participants classified themselves as following either a vegetarian or vegan diet (*n* = 45) than when we applied our strict classification criteria (Table 1) to their reported intake of animal and meat products (external classification). For our further analysis we used the external classification and 60.4% of participants followed an omnivorous (OM), 20.0% a vegetarian (VT) and 19.7% a vegan (VN) dietary pattern (Table 3) (*p* < 0.001).

**Table 3:** Comparison of self-reported and external dietary pattern of the survey participants in n (%); *n* = 2054

**dietary pattern**	**self reported**	**external classification**	*p*-value (test statistic, df)
**Omnivor**	1195 (58.18)	1240 (60.37)	< 0.001 (-4.85, 2043)
**Vegetarian**	436 (21.23)	410 (19.96)	0.022 (2.30, 2053)
**Vegan**	423 (20.59)	404 (19.67)	0.030 (2.17, 2053)

Significance was calculated with paired t-test

### Intake of supplements

Vegans were the most likely group to use a supplement (98.02%), followed by vegetarians (84.87%) and omnivores (67.33%) (*p* < 0.001) (Table 4). Dietary pattern was significantly associated with the use of dietary supplements even after adjusting for maternal age, education, household income, parity as well as awareness of the supplementation recommendations for lactating women (Table 5).

**Table 4:** Intake of supplements (multi- and mono- nutrient supplements) of the survey participants, by dietary pattern in n (%); *n* = 2054

	**Omnivore**	**Vegetarian**	**Vegan**	***p*-value (test statistic, df)**
**Intake of supplements in general**				
yes [n (%)]	835 (67.34)	348 (84.88)	396 (98.02)	0.001 (227.87, 2)
no [n (%)]	408 (32.66)	62 (15.12)	8 (1.98)	
Iodine^*^				
yes [n (%)]	686 (55.32)	245 (59.76)	211 (52.23)	0.092 (4.78, 2)
no [n (%)]	554 (44.77)	165 (40.24)	193 (47.44)	
DHA^*^				
yes [n (%)]	423 (34.11)	165 (40.24)	156 (38.61)	0.044 (6.23, 2)
no [n (%)]	817 (65.89)	245 (59.76)	248 (51.39)	
Vitamin B12^*^				
yes [n (%)]	677 (54.60)	253 (61.71)	235 (58.17)	0.033 (6.82, 2)
no [n (%)]	563 (45.40)	157 (38.29)		

* without inclusion of the best-case scenario; Significance was calculated with pearson’s chi-square test

**Table 5:** Relationships between dietary patterns and supplement intake, controlled for demographic variables and awareness of supplement recommendation

Independent variable	b	SE(b)	Wald	df	p	OR
Constant	-0.70	0.51	1.92	1	0.166	0.50
Age	0.02	0.02	2.41	1	0.121	1.02
Income^a^	0.12	0.12	1.00	1	0.317	1.13
Education^b^	0.33	0.12	7.64	1	0.006	1.40
First-time mother^c^	0.25	0.12	4.13	1	0.042	1.29
Awareness^c^	0.70	0.12	32.56	1	< 0.001	2.01
Vegetarian^c^	0.94	0.15	38.07	1	< 0.001	2.57
Vegan^c^	3.01	0.37	67.91	1	< 0.001	20.26

^a^ coded as 0 = income below median, 1 = income above median

^b^ coded as 0 = less than tertiary education, 1 = tertiary education

^c^ coded as 0 = no, 1 = yes; for categorical variables, 0 was set as the reference group

The composition of the three most frequently consumed dietary multi-nutrient supplements as well as their distribution among the dietary patterns are listed in Supplemental tables A3 and A4. Of all participants taking supplements, vegan participants most frequently (37.88%) reported taking mono-nutrient supplements followed by vegetarian (28.73%) and omnivorous (20.00%) participants. Women following a vegan diet frequently (24.26%) entered brand names of mono-nutrient supplements into the free-text field without specifying the respective product. To avoid possible underestimation of supplementation, best-case scenarios were developed based on the assumption that the reported free text field answers of brands translated to the intake of supplements each containing iodine, DHA or vitamin B12 respectively. However, this analysis is purely speculative. The frequency of reported mono-nutrients as brand names by vegetarian women was 7.32% and therefore we also give best case scenarios for this group as well while reporting of these brand names by omnivorous women is negligible (*n* = 19, 1.53%) and not shown.

### Iodine supplementation

There was no significant difference in iodine supplementation by dietary pattern (55.32% omnivores, 59.76% vegetarians and 52.23% vegans, Table 4). Assuming that iodine was taken through the unspecified mono-nutrient preparations, reported iodine supplementation would increase to 76.49% for vegans and 88.25% for vegetarians. The most frequently consumed dietary supplements contained 150 µg of iodine (Supplemental table A3).

### DHA supplementation

DHA supplementation was significantly different between the dietary groups (*p* < 0.05) (Table 4).Vegetarians (40.24%) were most likely to supplement DHA followed by vegans (38.61%) and omnivores (34.11%). In the best-case scenario DHA supplementation would increase to 62.87% for vegans and 47.56% for vegetarians.

DHA is recommended to all women with a fish consumption of less than two times a week. Our FFQ did not allow the resolution between once and twice a week and we thus provide the number only for less than or more/equal to once a week. In our study group only 27.94% of participants reported a fish consumption of equal to or more than once a week. We thus analyzed DHA supplementation by fish consumption. 72% of participants consumed fish less than once a week and therefore supplementation would be recommended. Of those, only 36.8% supplemented DHA while 63.2% did not (Table 6). The most frequently consumed supplements within our study contained 200 mg of DHA (Supplemental table A3).

**Table 6:** DHA supplementation and fish consumption in n (%), *n* = 2054

DHA supplementation	No or less than 1 time a week fish (%)	1 or more times a week fish (%)
**Yes**	545 (26.53)	199 (9.86)
**No**	935 (45.52)	375 (18.26)

### Vitamin B12 supplementation

Vegetarians reported the highest frequency of supplements containing vitamin B12 (61.71%) followed by vegans (58.17%) and omnivores (54.60%) (*p* < 0.05; Table 4). Assuming the best-case scenario 82.43% of vegans and 69.03% of vegetarians reported supplementation. The most commonly reported multi-nutrient supplements contained between 4.5 µg and 9 µg of vitamin B12 per daily dosage (Supplemental table A3). 2.9% participants reported intake of vitamin B12 in addition to a multi-nutrient (Supplemental table A5) with significant differences by dietary group. Here vegans reported the highest frequency of additional mono-nutrient intake (Supplemental table A5).

### Reported mono-nutrient supplements

Additionally, reported mono-supplements included vitamin D (9.8%), iron (4.8%), magnesium (3.2%), folic acid (3.1%), selenium (2.7%), calcium (1.7%), zinc (1.5%) and riboflavin (0.05%) (Supplemental table A6). There were significant differences by dietary group and except for zinc and riboflavin, vegans reported the highest frequency of intake (Supplemental table A6).

## Discussion

In Germany, there is a lack of data on the usage of dietary supplements among lactating individuals, and no research has yet examined the use of supplements based on dietary patterns such as omnivorous, vegetarian, and vegan. Our current study aims to fill this gap and we conducted an online study with 2054 participants including 410 vegetarian and 404 vegan lactating mothers. Our analysis shows that dietary pattern is an independent predictor of supplement use when adjusted for maternal age, parity, education, income and awareness of recommendations. Vegans reported the highest supplement usage, which is in line with previously published findings [46, 47]. We could further show that Vegans tended to consume single nutrient preparations (mono-preparations) more frequently while omnivores reported the use of multi-nutrient supplements more often which is consistent with previously published results [28]. Despite the high overall supplementation rates, our data indicate that a significant proportion of the study population did not adhere to the recommended supplementation of iodine and DHA as well as vitamin B12 for Vegans.

Adequate iodine intake is of general concern in the German population leading to the recommendation to supplement iodine during pregnancy and lactation [7]. Even in the best-case scenario 45% of omnivores, 12% of vegetarian and 24% of vegan lactating mothers and their infants are at risk for iodine deficiency. The low proportion of iodine supplement use in lactating mothers is in accordance with data from a 2009 survey of breastfeeding mothers in Germany where only 49% reported intake of iodine containing supplements [13]. Iodine use is recommended for all lactating mothers in Germany and has even been included in the official guidelines for pregnancy and postnatal care [48] yet there seems to be little progress in the rate of supplementation compared to 2009 [13]. One possible explanation is the low knowledge about the supplementation recommendation. Only about 40% of participants reported knowing the Germany Nutrition Society recommendation which is based on the “Healthy Start – Young Family Network” recommendation. Vegan participants most frequently indicating knowledge, followed by vegetarians and omnivores. This is in accordance with the general higher health awareness of vegans [49, 50]. It would be furthermore helpful that dietary supplements marketed for lactation contain exactly the recommended amount of iodine to avoid unnecessary confusion. The iodine concentration of the three most frequently consumed multi-nutrients was 150 µg which is, similarly to most products available for lactating mothers in Germany [51] above the recommendation of 100 µg/day by the Young Family Network [7]. The Tolerable Upper Limit (UL) of iodine intake during lactation is set at 600 µg/d by the European Food and Safety Agency (EFSA) [52] and it is thus unlikely that the current supplements lead to an intake of iodine above the UL. Manufactures are nonetheless encouraged to reformulate their products to meet the recommendations to avoid confusion on the side of the consumer.

DHA supplementation was reported by 63% of vegans and 48% of vegetarians when the best case scenario was taken into account [53]. DHA supplementation is only recommended when fish consumption is not adequate. Fish consumption was assessed by our mini FFQ (Supplemental table A1) which included the frequency 1–2 times a week, thus we cannot exactly quantify how many participants had an adequate fish consumption. Nonetheless, the overall fish consumption in our study was low and only 28% of participants reported weekly intake. Consequently, at least 72% of participants would meet the requirements of daily DHA supplementation, but only 36.8% of those reported DHA supplement use indicating significant room for improvement. The availability of microalgae oil as a source for DHA supplements makes a general supplementation recommendation to all mothers with inadequate fish consumption feasible, irrespective of dietary pattern, as it avoids the use of fish oil. Informing mothers of this possibility may be a good approach to boost the supplementation levels especially in those that do not consume fish due to ethical or environmental reasons. Furthermore, similar approaches as described for iodine could be employed to boost awareness of this supplementation recommendation. One first step would be the inclusion of DHA supplementation with inadequate fish consumption into the guidelines for pregnancy and postnatal care [48]. The dosage of most products marketed in Germany for lactation is in alignment with the recommendation made by the Young Family Network of 200 mg/day [51] and two out of the three most frequently cited multi-nutrient products (as listed in Supplemental table A3) contain exactly 200 mg. The EFSA has not established a tolerable upper intake level (UL) for DHA.

Multiple nutrients (B12, DHA/EPA, riboflavin, vitamin D, calcium, iron, iodine, zinc, selenium) can be critical during a vegan diet and in general a vegan diet during pregnancy and lactation is not recommended [21]. In this analysis we focused on vitamin B12 as this is the nutrient all vegans need to supplement due to its unreliable presence in vegan food sources. In our study 40% of vegans did not report the intake of vitamin B12 supplements. Even with the best-case scenario, where we counted ambiguous answers as a yes, the numbers are still high with 20% not supplementing vitamin B12. Our data is in contrast to previously reported data from breastfeeding vegans in America where high levels of maternal vitamin B12 supplementation (92%) were shown [54] as well as previously reported supplementation data for the general vegan population in Germany (92%) [55]. We cannot exclude that our low rate of supplementation is at least partially caused by reporting issues even though we tried to address them at least partially with our best-case scenario. Furthermore, even if vitamin B12 is supplemented, an adequate supply to mother and infant is not guaranteed. 17.2% of vegans and 31.6% of vegetarians reported the intake of B12 only through the top three multi-nutrient supplements (Supplemental tables A3 and A5). In these, vitamin B12 ranges between 4.5–9 µg. Estimates which take into account the absorption capacity of the body suggest that an intake of 50–100 μg B12 daily or 2000 μg weekly are needed for an adequate supply by supplements [56]. If the participants indeed did not take any additional vitamin B12 to their multi-nutrient preparation, the proportions of vegans and possible vegetarians with an inadequate supply of B12 may be even higher [55, 57]. Further studies should assess the actual vitamin B12 status of lactating mothers, especially those following a vegetarian or vegan diet. Overall, our results indicate a significant proportion of mothers that did not report intake of vitamin B12 or that may be taking inadequate amounts. Lactating mothers who do not supplement vitamin B12 on a strict plant-based diet, are at high risk of vitamin B12 deficiency with possible severe damage for themselves and their fully breastfed children. This is especially concerning as vegan mothers tended to fully breastfeed their children longer than the other dietary groups, which could increase the risk for deficiencies even further.

### Limitations

Participants were recruited online primarily through the use of social media and our findings may not be generalizable to the entire German population. Level of education and household income was higher than in the German general population. This might be explained by a higher health consciousness with higher socioeconomic status [58] and thus the greater interest in participating in such a health-related, voluntary survey. We specifically recruited vegan mothers and thus have an oversampling of this population compared to the general population in Germany. Furthermore, the online survey prohibited clarification questions and might thus underreport the intake of dietary supplements especially in the vegan group. The majority of the 396 vegans taking supplements made an entry in the free text field for supplement use (*n* = 303). This may result in a false-negative representation of dietary intake due to unspecific information. We tried to account for this possible underreporting by providing best-case scenarios under the assumption that the mentioning of two commonly stated brand names would translate to the respectively analyzed micronutrient. Additionally, we could not estimate the amount of the individual nutrient supplemented from our data as this was not reported for the mono-nutrient preparations.

## Conclusion

Dietary pattern is strongly associated with the use of dietary supplements during lactation with Vegans reporting the highest intake. Overall, however, our study suggests that the recommendation for iodine and DHA are not met by a high proportion of lactating mothers in Germany irrespective of the dietary patterns. Doctors, midwifes, pediatricians as well as other professions taking care of lactating mothers and their infants should be encouraged to inform mothers of the supplementation recommendations. Another strategy could be the use of social media (e.g., Instagram, YouTube) and social media influencers to spread awareness of this recommendation. Initiatives already in place, such as inclusion of the recommendation for iodine supplementation into the German guidelines for pregnancy and postnatal care [48] need to be updated to include recommendations for women following a vegetarian or vegan diet and need to be evaluated for their effectiveness. The guidelines should also be updated to include testing of vitamin B12 status in vegan and vegetarian mothers during pregnancy and lactation. The low intake of B12 supplements among vegan mothers requires further investigation. More research on which dietary supplements are used, especially in vegan and vegetarians with a special focus on the dosage of micronutrients needed to obtain an adequate nutrient status is urgently needed. In addition, it is important to conduct biochemical investigations to assess the micronutrient status of German mother-infant pairs who follow different dietary patterns, in order to ensure that both individuals receive adequate micronutrient intake during this crucial period.

### Supplementary Information

**Additional file 1**.

## Data Availability

Data and questionnaire are available upon request.

## Abbreviations

DHA: Docosahexaenoic acid
EPA: Eicosapentaenoic acid
FFQ: Food Frequency Questionnaire
OM: Omnivorous
SES: Socioeconomic status
VT: Vegetarian
VN: Vegan
