# Supplementary material for: Dietary supplement use among lactating mothers following different dietary patterns – an online survey
Source: Matern Health Neonatol Perinatol. 2024 Feb 1;10:3. doi: 10.1186/s40748-023-00171-3 (PMC10832081; doi:10.1186/s40748-023-00171-3)
Supplement: Supplementary file 1 — Additional file 1. [file 40748_2023_171_MOESM1_ESM.docx]

Dietary supplement use among lactating mothers following different dietary patterns– an online survey

**Additional Material**

**Table A1 – Mini food frequency questionnaire**

| Food | Frequency |
| --- | --- |
| Meat/ Sausages | - Never - less than once a week - 1-2 times a week - 3-4 times a week - 5-6 times a week - Once a day - Multiple times a day |
| Eggs |  |
| Fish |  |
| animal milk and animal milk products |  |
| Fruits |  |
| Vegetables |  |
| Meat substitute products |  |
| Plant based milk and milk products |  |
| Nuts |  |
| Pulses |  |
| Whole Grain products |  |

**Table A2 – List of supplements for selection in the questionnaire**

1. Abtei Vita Mama
2. Adfetal
3. Altapharma Schwangerschafts-Vitamine & DHA
4. Amitamin fertile F Phase 2
5. Avitale Folsäure 400 Plus
6. BabyForte Folsäure + Omega 3
7. BabyForte Folsäure Plus
8. Caneafem 2 Extrafolate-S + DHA
9. Caneafem 2 Extrafolate-S DHA mit Jod
10. Denk lactonatal + DHA
11. Denk prenatal + DHA
12. Doppelherz Mama plus mit DHA + Folsäure
13. Doppelherz System Schwangere + Mütter
14. Elevit 2 Schwangerschaft
15. Elevit 3 Stillzeit
16. Eucell Natal
17. Femibion 2 Schwangerschaft und Stillzeit
18. Femibion 3 Stillzeit
19. Femix omega
20. Femmoal Plus
21. Fetusan plus Omega-3
22. Folio Phase 2
23. Folio Phase 2 jodfrei
24. Gyn pregnant Maxi
25. Humana piùlatte
26. MensSana Multi für Schwanger + DHA
27. Mivolis Mama Folsäure + DHA
28. Orthomol Natal
29. Pure Encapsulations Schwangerschaftsformulierung
30. RedCare Folsäure + B12
31. Sana Expert Natalis lact
32. Sanct Bernhard Prenatal 2
33. Sanct Bernhard Folsäure-Jodid-Tabletten
34. Tetesept Femi Baby
35. Velnatal plus

**Table A3: Composition of the three most frequently consumed multi-nutrient preparations overall**

|  | **iodine**  **[µg]** | **DHA**  **[mg]** | **B12**  **[µg]** | **Folate**  **[µg]** | **B1**  **[mg]** | **B2**  **[mg]** | **B3**  **[mg]** | **B5**  **[mg]** | **B6**  **[mg]** | **B7**  **[µg]** | **E**  **[mg]** | **D**  **[µg]** | **A**  **[µg]** | **C**  **[mg]** | **Selenium**  **[µg]** | **Iron**  **[mg]** | **Zinc**  **[mg]** | **Magnesium**  **[mg]** | **Calcium**  **[mg]** | **Choline**  **[mg]** | **Luteine**  **[mg]** |
| --- | --- | --- | --- | --- | --- | --- | --- | --- | --- | --- | --- | --- | --- | --- | --- | --- | --- | --- | --- | --- | --- |
| 1 | 150,0 | - | 9,0 | 400,0 | - | - | - | - | - | - | - | 20 | - | - | - | - | - | - | - | - | - |
| 2 | 150,0 | 200,0 | 5,0 | 400,0 | 1,2 | 2,0 | 18,0 | 7,0 | 1,7 | 45 | 15,8 | 20 | 530 | 70 | 45 | 14 | 9 | - | 150 | 130 | 9,0 |
| 3 | 150,0 | 200,0 | 4,5 | 400,0 | 1,4 | 1,4 | 15,0 | 6,0 | 1,9 | 40 | 15,8 | 20 | - | 55 | 30 | 14 | 8 | 75 | - | - | 9,0 |

**Table A4: Most frequently used dietary multinutrient-supplements by dietary pattern in n (%); n=727.**

|  | all | omnivore | vegetarian | vegan | p-value |
| --- | --- | --- | --- | --- | --- |
| 1 | 404 (19.67) | 257 (20.73) | 86 (20.98) | 61 (15.10) | <0.001 |
| 2 | 214 (10.24) | 153 (12.34) | 41 (10.00) | 20 (4.95) | <0.001 |
| 3 | 109 (5.31) | 71 (5.73) | 18 (4.39) | 20 (4.95) | 0.026 |

Significance was calculated with pearson’s chi-squared test.

**Table A5: Simultaneous intake of DHA, iodine and vitamin B12 through mono-nutrient-supplements, list-supplements and other multi-nutrients from the free-text field in n and (%)**

| nutrient | all | omnivore | vegetarian | vegan | p-value |
| --- | --- | --- | --- | --- | --- |
| DHA | 13 (0.6) | 4 (0.3) | 2 (0.5) | 7 (1.7) | 0.007 |
| iodine | 31 (1.5) | 14 (1.1) | 6 (1.5) | 11 (2.7) | 0.077 |
| vitamin B12 | 59 (2.9) | 14 (1.1) | 14 (3.4) | 31 (7.8) | <0.001 |

Significance was calculated with pearson’s chi-squared test.

**Table A6: Mono-nutrients reported by dietary group in n (% of respective group)**

| nutrient | all | omnivore | vegetarian | vegan | p-value |
| --- | --- | --- | --- | --- | --- |
| iodine | 153 (7.5) | 51 (4.1) | 37 (9.0) | 56 (13.9) | p < 0.001 |
| DHA | 182 (8.9) | 36 (2.9) | 49 (11.9) | 97 (24.0) | p < 0.001 |
| vitamin B12 | 150 (7.3) | 15 (1.2) | 40 (9.8) | 95 (23.5) | p < 0.001 |
| riboflavin | 1 (0.05) | 1 (0.08) | 0 (0) | 0 (0) | n.s. |
| Vitamin D | 201 (9.8) | 70 (5.6) | 50 (12.2) | 81 (20.0) | p < 0.001 |
| folic acid | 63 (3.1) | 12 (1.0) | 17 (4.1) | 34 (8.4) | p < 0.001 |
| calcium | 34 (1.7) | 9 (0.7) | 8 (1.9) | 17 (4.2) | p < 0.001 |
| iron | 98 (4.8) | 36 (2.9) | 24 (5.9) | 38 (9.4) | p < 0.001 |
| magnesium | 66 (3.2) | 29 (2.3) | 15 (3.7) | 22 (5.4) | p < 0.05 |
| selenium | 55 (2.7) | 15 (1.2) | 10 (2.4) | 30 (7.4) | p < 0.001 |
| zinc | 30 (1.5) | 10 (0.8) | 12 (2.9) | 8 (2.0) | p < 0.001 |

Significance was calculated with pearson’s chi-squared test.
